# Determinants of health insurance enrolment in Sudan: evidence from Health Utilisation and Expenditure Household Survey 2009

**DOI:** 10.1186/1472-6963-14-S2-O17

**Published:** 2014-07-07

**Authors:** Isam Baloul, Maznah Dahlui

**Affiliations:** 1 Department of Social and Preventive Medicine (SPM), Faculty of Medicine, University of Malaya, Kuala Lumpur, Malaysia

## Background

Sudan established a National Health Insurance Fund (NHIF) in 1995. NHIF, despite the name, is a social health insurance scheme that predominantly enrols the formal sector workers, compulsorily, and the informal sector workers, voluntarily. The Government is targeting universal health coverage by 2031, by expanding the existing scheme. This paper aims to assess the insurance enrolment, and factors that determine such enrolment. Understanding these factors is important to ensure equity in acquiring healthcare and in identifying the barriers of its achievement.

## Materials and methods

Data used for this study were obtained from the Sudan Health Utilisation and Expenditure Household Survey conducted in 2009 (SHUEHS 2009). The survey was conducted at the nationwide level, covering 72,526 individuals of the 12,000 households. The Chi square test and bivariate were used to describe the characteristics of the insured and un-insured. Multivariate logistic regression was performed to identify factors explaining insurance enrolment.

## Results

Among a sample of 72,526 Sudanese, only 14,461 (19.9%) were insured. The enrolment showed regional and socio-economic disparities whereby, of the people living in the state capitals, 34% in Khartoum have insurance membership as compared to 14.3% in Darfur. Even after statistical adjustment, citizens living in Khartoum have almost twice the likelihood of insurance enrolment as those living in Darfur; OR 0.46 (95% CI 0.43-0.508). Moreover, rural-urban disparity was remarkable, 16.9% of the rural population are insured, compared to 25.3% of the urban dwellers. Urban have 26% higher enrolment likelihood compared to rural OR 1.26 (CI 95% 1.23-1.318). The well-off quintile had a 42% higher chance of being enrolled than the poorest; OR 1.429 (95% CI 1.34-1.52). As expected, the enrolment was determined by occupation, as civil service workers had an 80% higher chance of being enrolled, compared to those unemployed. People with a university and higher education background were more likely to be insured, compared to those without formal education. People with diabetes and hypertension were more likely to be insured, than those without such conditions OR 1.25(95% CI 1.06-1.48), 1.31(95% CI 1.16-1.502), respectively.

## Conclusions

From the SHUEHS 2009 data, it is evident that there were regional and socioeconomic disparities for the NHIF enrolment. Policies and interventions to close the disparities are an urgent need.

